# Optimization and validation of an HS-SPME/GC-MS method for determining volatile organic compounds in dry-cured ham

**DOI:** 10.3389/fnut.2024.1342417

**Published:** 2024-02-01

**Authors:** Katja Babič, Lidija Strojnik, Andrija Ćirić, Nives Ogrinc

**Affiliations:** ^1^Jožef Stefan International Postgraduate School, Ljubljana, Slovenia; ^2^Department of Environmental Sciences, Jožef Stefan Institute, Ljubljana, Slovenia; ^3^Department of Chemistry, Faculty of Science, University of Kragujevac, Kragujevac, Serbia

**Keywords:** dry-cured ham, HS-SPME/GC-MS, optimization, response surface methodology, validation

## Abstract

The formation of volatile organic compounds (VOCs) in dry-cured ham is a result of different biochemical and enzymatic processes. Moreover, accurately quantifying these VOCs is challenging since ham is a complex matrix, which contains compounds from various chemical families and a wide range of volatilities of different molecular masses. In this study, we systematically optimized and validated an analytical method for quantifying VOCs in dry-cured ham using headspace solid phase microextraction (HS-SPME) coupled with gas chromatography-mass spectrometry (GC-MS). Optimal SPME conditions were determined through both an experimental procedure (one-factor-at-a-time) and response surface methodology (RSM), revealing that a 60-min equilibration at 70°C, a 60-min extraction at the same temperature, and a 4-min desorption time at 250°C provided the most favorable results. To enhance quantitation, twelve multiple internal standards (ISTDs) were employed to address and improve the quantitation of the 12 VOCs. Method validation covered aspects of linearity, limits of detection (LOD: 0.03–1.13 mg kg^−1^), limits of quantitation (LOQ: 0.09–3.41 mg kg^−1^), and working ranges (0.01–19.1 mg kg^−1^). The practical application of this optimized method was demonstrated by analyzing dry-cured ham samples (*n* = 4), sourced from the Slovenian market. The initial statistical evaluation indicates that different types of dry-cured hams can be differentiated (with an 83.1% of accuracy) according to their aromatic profile. However, a larger sample size would be required to provide a more comprehensive assessment.

## 1 Introduction

The analysis of volatile organic compounds (VOCs) has been used to characterize dry-cured hams regarding their unique flavor, economic value, consumer experience, and long-term preservation (1). In Europe, particularly in the Mediterranean region, various hams with Protected Designation of Origin (PDO) status, including Spanish Serrano and Iberian hams, Italian San Daniele, Toscano, and Parma, as well as French Bayonne hams, are among the most important products in their respective countries (1, 2). *Kraški pršut*, a traditional Slovenian-Mediterranean dry-cured ham with Protected Geographical Indication (PGI), is significant in Slovenia's culinary heritage and is the country's most recognized and appreciated meat product (3). Dry-cured ham is produced using a traditional method involving dry salting, long ripening time, and the absence of smoking, resulting in a unique flavor and texture (4). Its distinctive aroma originates from the raw material and the presence of various volatile compounds formed mainly through a several enzymatic and non-enzymatic reactions during ripening (1, 5–7). Furthermore, biochemical processes like lipolysis and proteolysis contribute to developing various VOCs and their precursors (2, 5), including aldehydes, alcohols, ketones, esters, hydrocarbons, and acids (2, 6). The analysis of VOCs provides valuable information about dry-cured hams' sensory quality (1, 5), a significant factor affecting consumer purchasing decisions (7).

A major advancement in the analysis of VOCs has been introduced by headspace-solid phase microextraction (HS-SPME), developed by Pawliszyn and colleagues in the 1990s (8, 9). This technique is fast, accurate, rapid, sensitive and solvent-free (8, 10–15). Its combination with gas chromatography-mass spectrometry (GC–MS) has proven highly efficient for sampling and analyzing volatile compounds of meat and meat products (2, 8, 11). Despite its many advantages, optimizing SPME for VOC analysis is crucial since many parameters, including equilibrium time, extraction time, and temperature, can significantly affect extraction efficiency (1, 16, 17). Conventional parameter optimization, involving a one-factor-at-a-time approach, can be arduous and lengthy (18). Therefore, response surface methodology (RSM) has emerged as an alternative optimization strategy. RSM is a statistical approach for experimental design implemented in mathematical modeling. It enables the assessment of the influences of various factors and their interactions on one or more response variables with fewer experimental measurements (19). It is a powerful approach widely used in many applications (20).

Method validation is closely linked to method development and includes determining linearity, precision, range, detection limit, quantification limit, and robustness (21). Nevertheless, in instance where quantification is the objective, various measures are necessary to garantee an unbiased quantification process. In HS-SPME that relies on the equilibration of compound and fiber, incorporating appropriate internal standards (ISTDs) becomes indispensable for effective peak area normalization. The reason is that these ISTDs consider for the differences resulting from variations in the absorption capacity of different fibers, fiber wear, competition among different molecules with varying affinities for the sorbent, and changes in sorption temperature among different samples. Using an appropriate ISTD also allows for the correction of deviations from linearity, and, in some instances, an individual ISTD can be used for data normalization during quantification (22). As reported by Fortini et al. (22), to achieve this objective, the following steps are necessary: selecting appropriate internal standards for each identified volatile compound and expanding the linear working range for each one identified volatile compounds to encompass both lower and higher concentrations.

This paper presents the optimization and validation of a HS-SPME/GC–MS method for extracting VOCs from dry-cured ham. It encompasses the following aspects: (i) optimization of SPME conditions using both one-factor-at-a-time and RSM approaches; (ii) method validation; (iii) selection of appropriate internal standards and validation based on multiple ISTD normalization to enhance the quantification of VOCs in dry-cured ham, and (iv) a preliminary investigation of dry-hams available in the Slovenian market.

## 2 Materials and methods

### 2.1 Chemicals and reagents

Pure chemicals: 1-octanol; 1-octen-3-ol; hexanal; heptanal; octanal; nonanal; 2-decenal, *(E)*; benzaldehyde; hexanoic acid; octanoic acid; dodecanoic acid; benzene, 1,4-dimethoxy; 2-hexen-1-ol, *(E)*; 2-hexen-1-ol, acetate, *(E)*; 3-heptanone, 2-methyl; acetic acid, pentyl ester; acetic acid, hexyl ester; butanoic acid, methyl ester; butanoic acid, ethyl ester; butanoic acid, butyl ester; hexanoic acid, ethyl ester; linalool; γ-dodecalactone and toluene-d_8_ were purchased from Sigma-Aldrich (Madrid, Spain). The analytical standards *n*-alkanes mixture (C_10_–C_40_) for retention index (RI) assessment determination was supplied by Supelco (St. Louis, USA).

### 2.2 GC-MS analysis

#### 2.2.1 Samples and standard mixtures preparation

An external standard mixture, comprising 12 compounds: 1-octanol; 1-octen-3-ol; hexanal; heptanal; octanal; nonanal; 2-decenal, *(E)*; benzaldehyde; hexanoic acid; octanoic acid; dodecanoic acid, and benzene, 1,4-dimethoxy, was prepared. These compounds were chosen based on the VOCs obtained from the analysis of selected dry-cured hams (3, 4, 23). The whey protein/oil mixture was used to mimic the meat matrix, specifically protein and fat components. The mixture was prepared according to the experience proposed in the literature (22, 24). Briefly, 5 mg of each compound was weighed and incorporated into a matrix, consisting of unflavoured whey protein (Me:First) and ICP-MS vacuum pump oil (Agilent) in a 3:1 ratio. The whey protein/oil blend was prepared immediately before analysis and tested to be free from distinctive VOCs. This external solution mixture was employed for method validation, encompassing linearity, limits of detection and quantification, recovery, and the working range.

A mixture of internal standards (ISTD MIX) was prepared and added to both samples and calibrants for quantification. This mixture comprises 12 compounds: 2-hexen-1-ol, *(E)*; 2-hexen-1-ol, acetate, *(E)*; 3-heptanone; 2-methyl; acetic acid, pentyl ester; acetic acid, hexyl ester; butanoic acid, methyl ester; butanoic acid, ethyl ester; butanoic acid, butyl ester; hexanoic acid, ethyl ester; linalool; γ-dodecalactone and toluene-d_8_. The selection of these compounds was based on their non-presence in dry-cured ham samples (23) and their ability to provide a range of low and high molecular mass ISTD compounds for various compound classes (22). Considering their chemical properties, the peak areas of these compounds were used for area normalization when constructing calibration curves for the target compounds (22). Each ISTD in the mixture was at a final concentration of 50 mg kg^−1^. All standards, including the ISTD MIX, were stored in the fridge at −20°C.

Samples of dry-cured ham, including PGI Kraški pršut (K1); dry-cured ham from Kraškopolje pig, which is only preserved Slovenian autochthonous pig breed (KP); dry-cured ham made from pork of the Mangalica variety (M1), and Spanish Jamon Serrano (JS) were purchased in the Slovenian market. A known weight (1 g) of homogenized sample, previously frozen with liquid nitrogen was ground and transferred to a SPME glass vial (10 mL), followed by the addition of 1 mL of a saturated NaCl solution and 50 μL of the internal standard solution ISTD MIX.

#### 2.2.2 Headspace solid-phase microextraction

The SPME extraction procedure was performed using a Divinylbenzene/Carboxen/Polydimethylsiloxane (DVB/CAR/PDMS) SPME fiber (2 cm × 50/30 μm thickness) purchased from Sigma-Aldrich (Supelco, Bellefonte, USA). The SPME fiber was pre-conditioned at 270°C for 30 min before the first use. Before each extraction, the SPME fiber was conditioned for 5 min at 250°C before analysis and for 20 min at 250°C after analysis. For each sample, 1.0 g of the standard solution was weighed into a 10 mL SPME glass vial (Supelco), followed by 1 mL of a saturated NaCl solution and 50 μL of the ISTD mix. The same procedure was replicated for the dry-cured ham samples. The vials were tightly capped with a silicone/PTFE septum. The volatiles were then extracted under optimal HS-SPME conditions, which included a 60-min equilibration time and a 60-min extraction time, both at a temperature of 70°C. Afterwards, the analytes were desorbed for 4 min in the GC injector (250°C) equipped with a straight Ultra Inert Solid Phase Microextraction Liner (Sigma-Aldrich, Supelco, USA) operating in splitless mode. Each sample was analyzed in duplicate.

#### 2.2.3 Gas chromatography-mass spectrometry

All analyses were performed using a 7890B Gas Chromatograph and a 5977A Series Gas Chromatograph/Mass Selective Detector (Agilent Technologies, Santa Clara, USA). The separation process was accomplished using a high-performance VF-WAXms polyethylene glycol column (30 m × 0.25 mm × 0.25 μm, Agilent J&W, USA). Helium was used as the carrier gas at a constant flow rate of 1.5 mL min^−1^. The oven temperature program was selected according to the literature data (7, 25–28), additionally optimized on dry-cured ham samples and set in the following way: 40°C (held for 1 min) to 150°C at 6°C min^−1^, to 200°C at 10°C min^−1^, and to 250°C at 20°C min^−1^ (held for 10 min), resulting in a total analysis time of 37 min. The quadrupole, interface, and ion source temperatures were set at 180, 280, and 240°C. Electron ionization (EI) was performed at 70 eV with an *m/z* scan range of 35–300 at a scan rate of 5.2 scans s^−1^ (Full scan mode).

Data were acquired using ChemStation software (Agilent, USA). Compound identification was conducted through (i) spectral similarity search in the NIST Spectral Database 14 (National Institute of Standards and Technology, Gaithersburg), (ii) matching retention times with available standards, and (iii) matching the calculated retention indices (RIs) using the Van Den Dool and Kratz equation (29) with the accessible RIs found in the NIST Chemistry WebBook, SRD 69, and PubChem database (specifically for normal alkane RI, polar column, custom temperature program) (26).

The HS-SPME/GC-MS method was validated for 1-octanol; 1-octen-3-ol; hexanal; heptanal; octanal; nonanal; 2-decenal, *(E)*; benzaldehyde; hexanoic acid; octanoic acid; dodecanoic acid, and benzene, 1,4-dimethoxy. Quantification was performed through at least a five-point linear least squares calibration of the analyte peak area relative to the internal standard peak (area ratio) plotted against the analyte concentration ratio (amount ratio).

### 2.3 Response surface methodology

The Box-Behnken design of experiments, which enables modeling of the response surface (Design Expert trial version 9.0 software, State-Ease, Inc., Minneapolis MN, USA), was used to investigate the effect of four factors: equilibration time (20, 30, 40, 50, 60 min), equilibration temperature (40, 50, 60, and 70°C), extraction time (20, 30, 40, 50, 60 min), and extraction temperature (40, 50, 60, and 70°C). In addition, Response surface methodology (RSM) with central composite design (CCD) was used to optimize the extraction parameters, including the concentration of ethanol (X_1_), incubation temperature (X_2_), and solvent-to-solid ratio (X_3_). The range and central point values of the three process variables are presented in Supplementary Table 1. The process variables were coded according to the following equation:


(1) $$\begin{array}{l} x=\frac{X_{i}-X_{0}}{\Delta X} \end{array}$$

where *x* is the dimensionless coded value, *X*_*i*_ is the actual value of variables, *X*_0_ is the actual value of variables at the center point, and Δ*X* is the step change value. The data were fitted with a second-order polynomial equation as follows:


(2) $$\begin{array}{l} Y=\beta_{0}+\overset{n}{\underset{i=1}{\sum }}\beta_{ii}X_{i}+\overset{n}{\underset{i=1}{\sum }}\beta_{ii}X_{i}^{2}+\overset{n-1}{\underset{i=1}{\sum }}\overset{n}{\underset{j=2}{\sum }}\beta_{ii}X_{i}X_{j}+\varepsilon \end{array}$$

where *Y* is the predicted responses (AVE, ALD, FA), β_0_ is the model constant, β_*i*_, β_*ii*_ and β_*ij*_ are model coefficients (linear, squared and interactive effects), and ε is the error (19).

### 2.4 Data analysis

Statistical analysis included one-way ANOVA and F-test. Probability (*p*)-values of lower than 0.05 were used to indicate a significance level. Data visualization was performed in OriginPro 2021 (OriginLab Corporation, Northampton, MA, USA). Principal component analysis (PCA) was used on real samples to classify samples based on the aroma profiles. The analysis was performed using the XLSTAT software package (Addinsoft, New York, USA).

## 3 Results and discussion

### 3.1 Optimization of the HS-SPME conditions

The default fiber for the HS-SPME procedure was DVB/CAR/PDMS, recognized for its capability to extract volatile and semi-volatile compounds and cover an extensive array of molecular weights (30). The optimization of extraction conditions considered parameters such as incubation temperature (ranging from 40 to 70°C), equilibration time (20–60 min), extraction time (20–60 min), and desorption time (1–5 min). The selected optimal conditions are described in Table 1. Similar conditions were also obtained in several applications including essential oils, fruits, truffles (15, 25–27, 31), which indicates the general usefulness of the optimized method. However, it needs to be tested or adapted for each specific matrix.

**Table 1:** Optimization using one-factor-at-a-time test.

	**Equilibration time (min)**	**Equilibration temperature (°C)**	**Extraction time (min)**	**Extraction temperature (°C)**
1	20	40	20	40
2	40	50	40	50
3	60	60	60	60
4		70		70

#### 3.1.1 Optimization using one-factor-at-a-time approach

##### 3.1.1.1 Equilibration and extraction temperature

**Figure 1:**
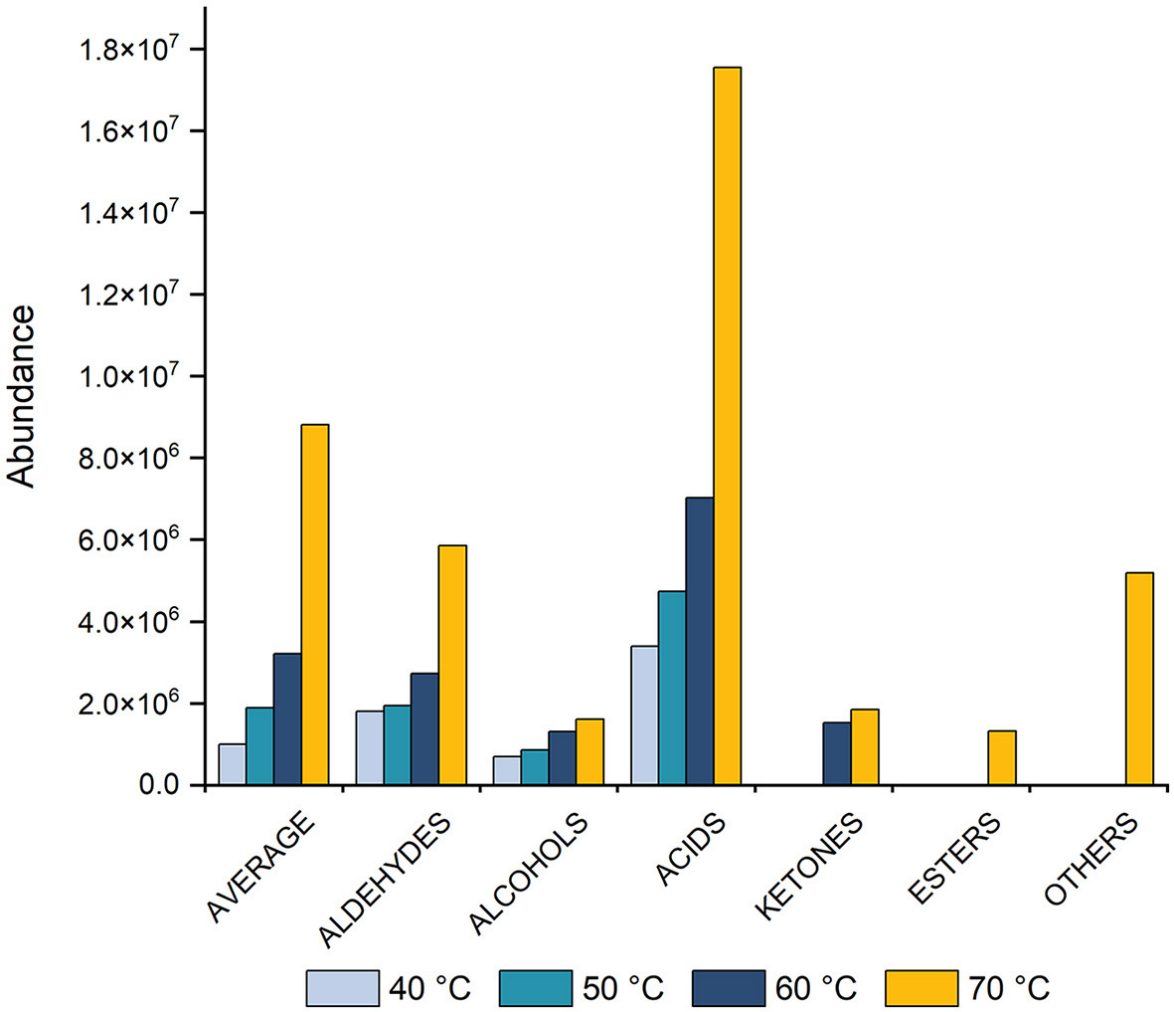
Histogram showing the effect of equilibration and extraction temperatures (40, 50, 60, and 70°C) on the average peak area for total VOCs, including aldehydes, alcohols, acids, ketones, esters, and other compounds. Measurements were taken after 60 min of equilibration and 60 min of extraction, followed by a 4-min desorption process.

To assess the impact of temperature on average signal intensity (peak area), four temperatures: 40, 50, 60, and 70°C were tested. The results revealed that increasing the temperature led to a corresponding increase in peak area (Figure 1). The optimal extraction and equilibration temperature were determined to be 70°C. Notably, at 70°C, the aromatic profile remained unchanged, with no formation of new compounds or degradation of existing ones. Compared to a lower temperature (e.g., 40°C), the extraction process occurs more rapidly at a higher temperature (70°C), thereby obtaining higher concentrations of compounds. Previous studies have reported extraction temperatures ranging from 40 to 60°C (1, 5, 10, 32–34), and in some instances, even higher temperatures, such as 73°C have been used (34). However, the extraction temperature used in these studies had not been optimized.

##### 3.1.1.2 Equilibration and extraction time

**Figure 2:**
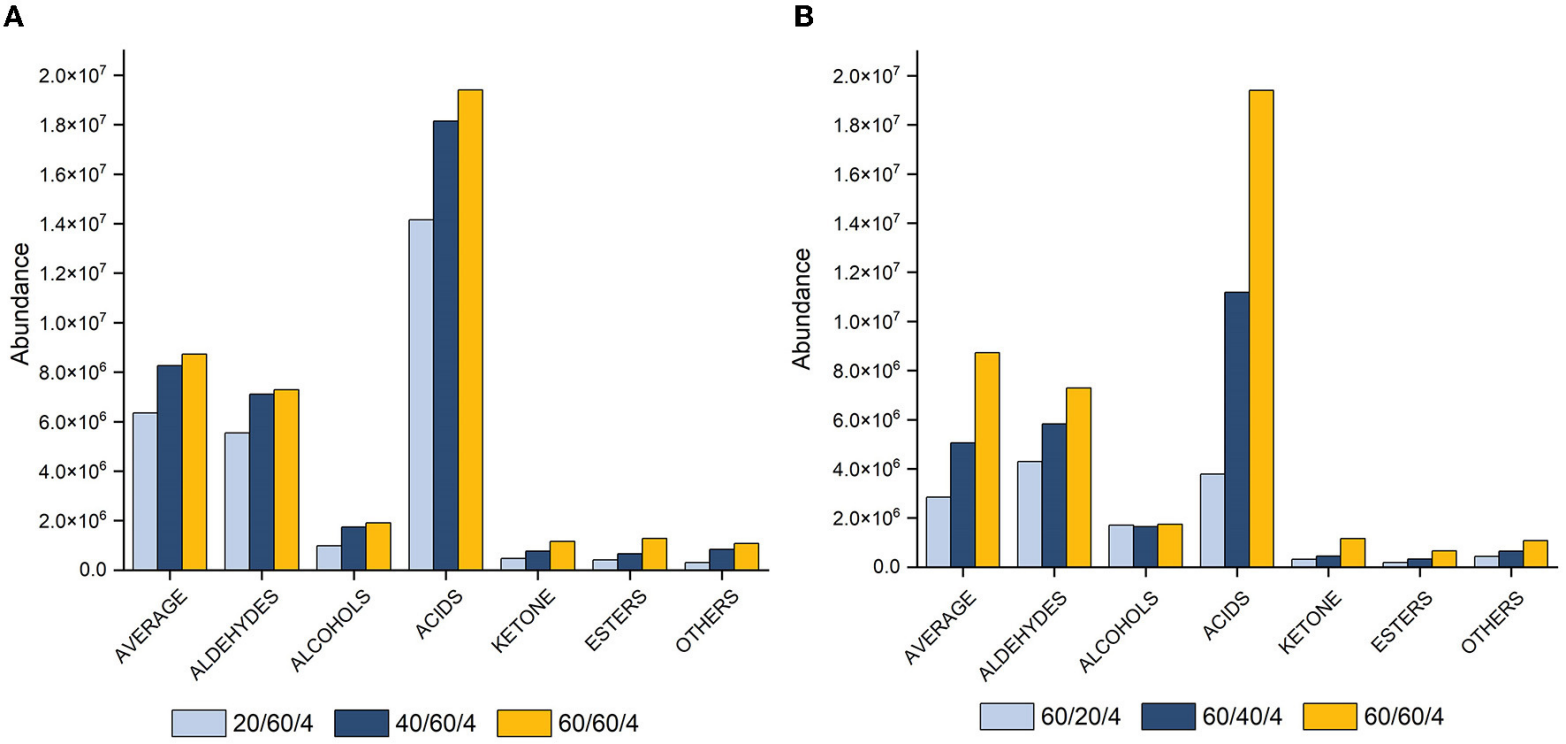
Histograms showing peak area with varying equilibration **(A)** and extraction **(B)** times (20, 40, and 60 min) at 70°C and 4 min of desorption for total VOCs, aldehydes, alcohols, acids, ketones, esters, and other compounds.

The samples were analyzed at different equilibration times (20, 40, and 60 min) and extraction times (20, 40, and 60 min) at an optimal temperature of 70°C, with a desorption time of 4 min. The results revealed that longer equilibration and extraction times increased peak area (Figure 2), while shorter equilibration and extraction times (20 min) extracted fewer compounds, especially fatty acids. The highest extraction yield was observed at 60 min for all compound classes (Figure 2B). Consequently, a combination of 70°C and an extraction time of 60 min was determined as optimum and used for method validation.

##### 3.1.1.3 Desorption temperature and time

**Figure 3:**
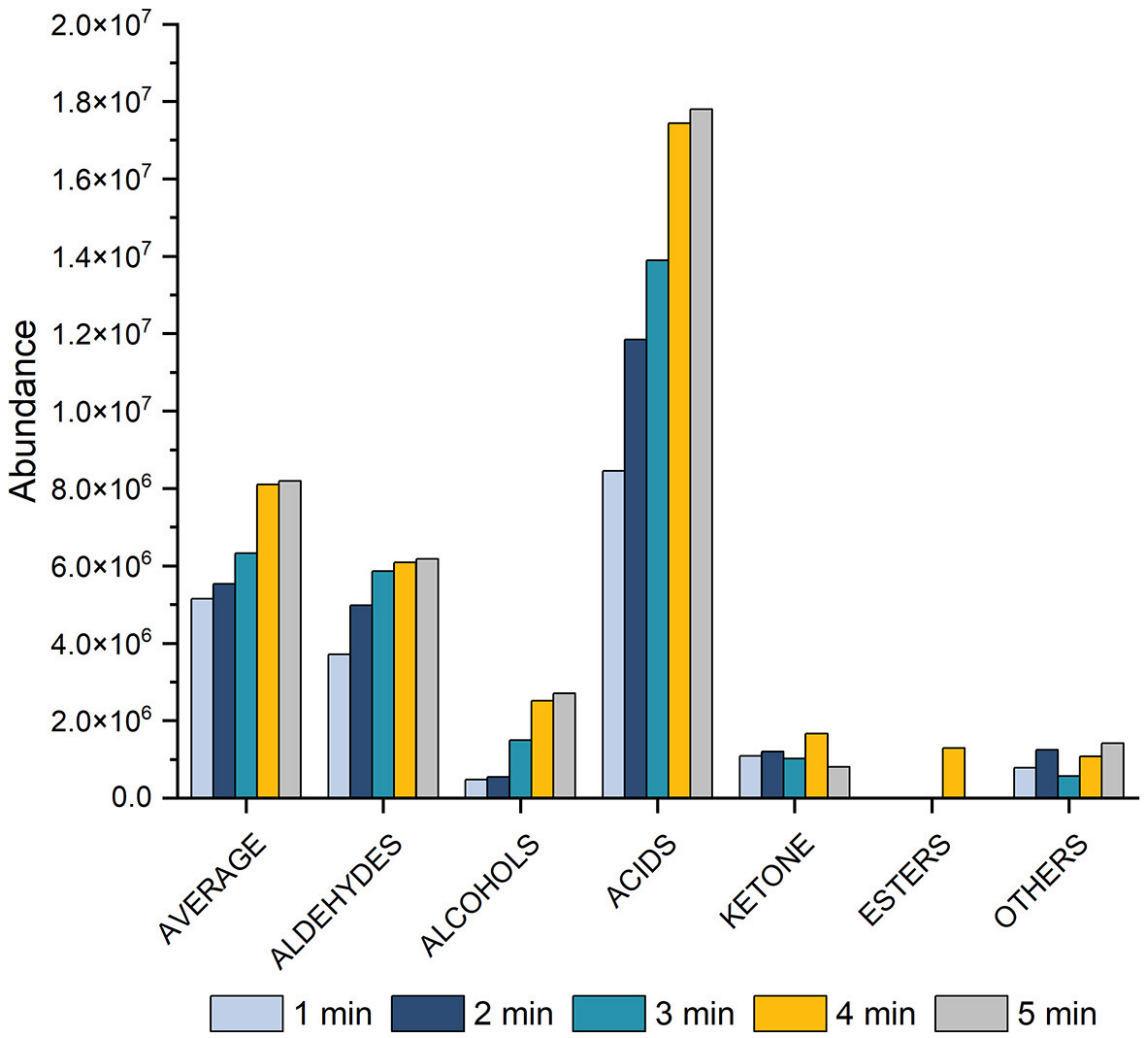
Histogram showing the effect on peak area of varying the desorption time (1, 2, 3, 4, and 5 min) at 60 min of equilibration, 60 min of extraction at 70°C equilibration for total VOCs, aldehydes, alcohols, acids, ketones, esters, and other compounds.

We assessed various desorption times (1, 2, 3, 4, and 5 min) for VOCs, taking into account the time intervals between SPME fiber injection into the GC port. The results indicated different effects of desorption time on the acid class compared to other compound classes (Figure 3). A 5-min desorption time gave the best results for acids, while for other chemical classes, the optimal was 4 min. Based on these findings, a desorption time of 4 min was finally chosen as the optimal time.

##### 3.1.1.4 Salt effect

Adding salt, typically sodium chloride (NaCl), to the samples elevates the solution's ionic strength (27), which in turn decreases the solubility of the analytes and amplifies their volatility. Consequently, the enhanced volatility leads to greater adsorption of the analytes onto the fiber, thereby improving the extraction efficiency (35). Initially, VOCs were analyzed in the homogenized sample, and the obtained results were subsequently compared between samples with (a) added deionized water and samples with (b) added a saturated NaCl solution. The findings indicated that adding a saturated NaCl solution improved the extraction of volatiles.

#### 3.1.2 Response surface methodology

Based on the preliminary screening experiments, the following independent variables were selected: equilibrium time (factor X_1_: 20, 40, 60 min), extraction time (factor X_2_: 20, 40, 60 min), extraction temperature (factor X_3_: 50, 60, 70°C) and desorption time (factor X_4_: 1, 2, 3, 4, 5 min). Boundary values for factors X_1_, X_2_, and X_3_ were determined through screening experiments. Factor X_4_ (desorption time) was specifically investigated to understand its impact on the extraction rate. Consequently, its boundary values were set between 1 and 5 min, aligning with the screening experiment time range, as no significant enhancement in the extraction rate was evident beyond this interval. The experimental design utilized a Box-Behnken design, comprising 15 runs, each with corresponding responses (36). In addition, a three-factor inscribed central composite design was utilized to uncover the relationships between the response functions and the process variables (Equation 1) (37). The extraction conditions for aldehydes and acids were simultaneously optimized. Supplementary Table 1 presents the results of the empirically measured responses data for the 15 runs in accordance with the experimental design. The yield of the total aldehyde varied from 0.164 × 10^6^ to 5.328 × 10^6^, while that of acids ranged from 0.528 × 10^6^ to 19.11 × 10^6^. The highest yield was achieved during the 11th run under the experimental conditions of X_1_ = 60 min, X_2_ = 60 min, and X_3_ = 70°C. Informed by these findings, the extraction procedure was refined to maximize the response outcomes.

##### 3.1.2.1 Fitting the model

The analysis of variance (ANOVA) confirmed the adequacy of the model in representing the relationship between the response variable and independent variables (36). The close correspondence between experimental and predicted values suggests a satisfactory model (19). Coefficients of the predicted model in coded variables, concerning average aromatic profile content (AVE), aldehydes, and acids, were analyzed for significant contributions using the *p*-value of the *F*-test (*p* < 0.05). The model's fitness was further evaluated through the lack-of-fit test (*p* > 0.05) (19). The coefficient of determination (*R*^2^) served as an additional measure of fit quality, yielding values of 0.9173, 0.7938, and 0.8967 for the AVE, aldehydes, and acids, respectively. The model's significant adequacy was confirmed at a 0.0001% probability level, with both *R*^2^ and adjusted *R*^2^ exceeding 70%. Additionally, no evidence of lack of fit was observed for the model in all responses, indicated by a *p*-value > 0.05 (19). The final equations obtained when optimizing the experimental conditions and performing the ANOVA are presented in Table 2.

**Table 2:** Final equations (Equation 2) obtained when optimizing the experimental conditions and performing the ANOVA.

**Responses**	**Regression model**	***R* ^2^**	**Adj. *R*^2^**	**Adeq. precision**
Y_1_ (AVE) =	5.322 × 10^13^ + 1.435 × 10^13^X_2_ + 3.125 × 10^13^X_3_ + 7.627 × 10^12^ X_2_X_3_	0.9173	0.8335	17.384
Y_2_ (ALD) =	2.574 × 10^13^ + 1.148 × 10^13^X_2_ + 6.349 × 10^12^X_3_ + 3.519 × 10^12^${\text{X}}_{2}^{2}$ + 4.394 × 10^12^X_2_X_3_	0.7938	0.7113	9.391
Y_3_ (FA) =	3.500 × 10^14^+ 3.588 × 10^9^X_1_ + 5.852 × 10^13^X_2_ + 2.424 × 10^14^X_3_ + 1.608 × 10^13^X_1_X_2_ + 3.308 × 10^13^X_2_X_3_	0.8967	0.8397	13.084

The adequate precision value (adeq precision) is also presented representing the signal-to-noise ratio. All values are >4 indicating their adherence to the desirable criterion.

X_1_, equilibrium time (min); X_2_, extraction time (min); X_3_, extraction temperature (°C), X_4_, desorption time (min). Level of significance *p* < 0.001.

##### 3.1.2.2 Effect of extraction variables on average aromatic profile content

The model showed a high significant correlation with the experimental data (*p* < 0.001). An ANOVA indicated significant linear (X_2_ and X_3_) and interactive (X_23_) effect on the average (AVE) content (*p* < 0.001). Examining the regression coefficient (β) values, extraction temperature (X_3_) demonstrated a significant positive impact, followed by extraction time (X_2_). Upon removing non-significant variables and refitting the second-order polynomial, the lack of fit's non-significant value (*F* = 4.14) (38) suggested that the model effectively captures the spatial influence of variables on the response, providing reliable predictions (*R*^2^ = 0.9173). The AVE content exhibited a significant increase (*p* < 0.001) as the equilibrium time extended from 20 to 60 min. This result can be explained by mass transfer principles, with the concentration gradient between solid/solvent—head space—fiber described in detail in Souza-Silva et al. (39).

**Figure 4:**
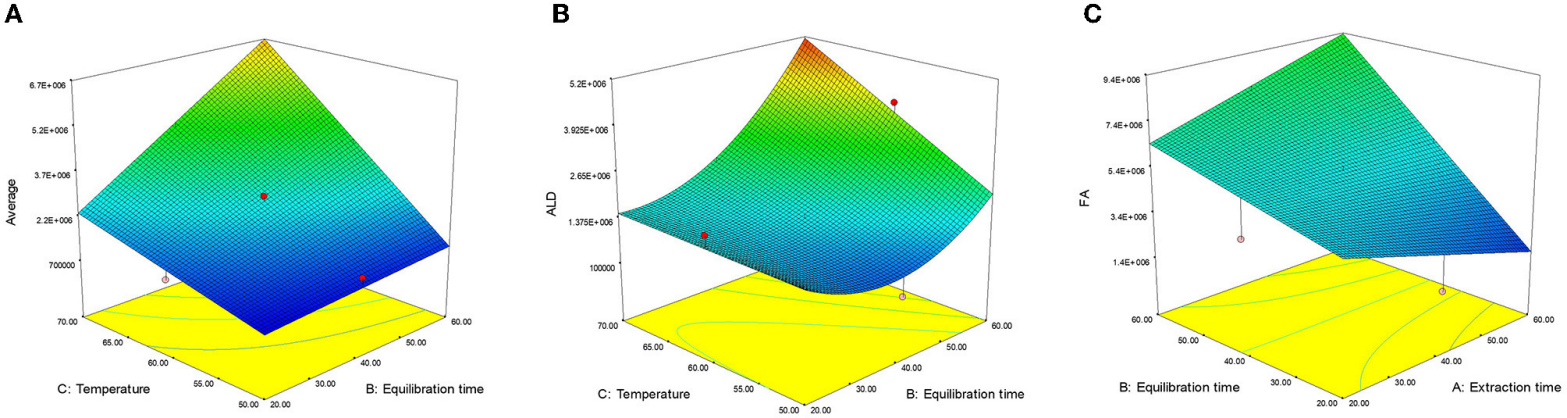
Effect of extraction variables on **(A)** average (AVE), **(B)** aldehydes (ALD), and **(C)** fatty acids (FA).

Likewise, we observed a significant positive effect (*p* < 0.001) of solvent concentration and extraction temperature on the levels of aldehydes (ALD) and fatty acids (FA). Regarding the interactive effect of the variables, only the interaction between extraction time and temperature (X_23_) on the AVE content was significant (*p* < 0.001). With an increase in equilibrium time and extraction temperature, AVE content also significantly increased (Figure 4A). The increased solubility of AVE content with higher extraction temperature likely results from the elevated temperature and extended equilibrium time, leading to improved enhanced mass transfer between solid/solvent—head space—fiber.

##### 3.1.2.3 Effect of extraction variables on total aldehyde

The linear effect of extraction time (X_2_) and extraction temperature (X_3_) along with their interaction (X_23_), positively influenced the ALD content. The ALD content exhibited a strong dependency on extraction time (X_2_), followed by extraction temperature (X_3_), the interactive effect (X_23_), and the quadratic of extraction time (${\text{X}}_{2}^{2}$), as supported by their regression coefficient (β) values. The non-significant lack of fit value (*F* = 2.57) (38) indicated that the model was well-fitted with good precision (*R*^2^ = 0.7938). Interactions between X_2_ and X_3_ had a significant (*p* < 0.05) positive impact on ALD content, which further increased with longer equilibrium time (X_2_) and higher extraction temperature (X_3_). This interaction effect was particularly pronounced at higher values of both variables (Figure 4B).

##### 3.1.2.4 Effect of extraction variables on fatty acids

The FA content was significantly influenced by various factors, including linear effects (X_1_, X_2_, and X_3_) and interactive effects (X_12_ and X_23_). Extraction temperature (X_3_) produces a maximum effect on FA content, followed by extraction time (X_2_), interactive effects (X_23_), and (X_12_) as confirmed by their *R*^2^ (β) values. Following the removal of non-significant factors and fitting a second-order polynomial equation, the non-significant lack of fit value (*F* = 3.28) (38) showed that the model is fitted with good precision (*R*^2^ = 0.8967). The response surface 3D graph for FA content is shown in Figure 4C. The interaction effect of equilibration and extraction time (X_12_) demonstrated a significant (*p* < 0.01) positive impact on FA content. At lower values of X_1_ and X_2_, FA content increased, but as both values increased, the interactive effect (X_23_) on FA content become predominant (Figure 4C). The interaction of extraction time and temperature (X_23_) also exhibited a significant (*p* < 0.001) positive effect on FA content.

##### 3.1.2.5 Validation of the predictive model

The software forecasted the optimal values for the independent variables: equilibrium time, extraction time, and extraction temperature to be 60.0 min, 60.0 min, and 70°C, respectively, while the predicted total aldehyde concentration (Abundance) was 5.157 × 10^6^ and 1.6304 × 10^7^ for fatty acids. Compared with the software (Design-Expert), the predicted results are closely aligning with the actual results, suggesting that the optimization parameters put forward in this study are accurate.

**Figure 5:**
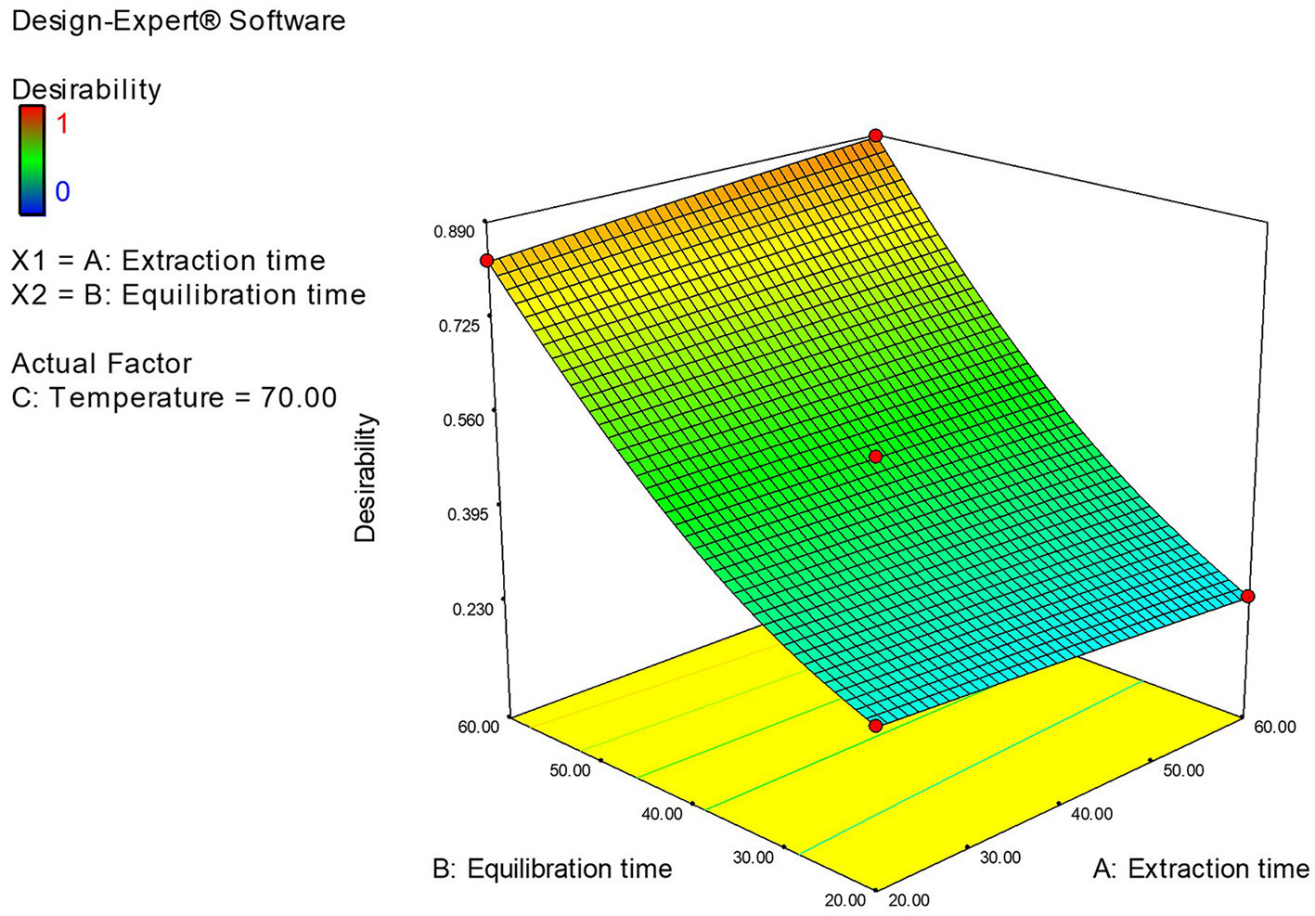
Design-experiment model showing the optimum values for the independent variable equilibrium time, extraction time, and extraction temperature to be 60 min, 60 min, and 70°C.

The optimization of the critical variables in the HS-SPME/GC-MS was conducted using both the RSM and experimental (one-factor-at-a-time) approaches. The main variables included equilibrium time (20–60 min), extraction time (20–60 min), and extraction temperature (40–70°C). Based on the results obtained from both methods, the optimal SPME conditions were 60 min of equilibration, 60 min of extraction at 70°C, and a desorption time of 4 min. Results of the Design-experimental model is presented in Figure 5.

### 3.2 Method validation

Method validation using the defined optimal HS-SPME parameters is summarized in Table 3. The regression coefficients ranged from 0.992 to 0.999, and the adjusted regression coefficients were >0.992 (0.01–19.1 mg kg^−1^). However, while the correlation coefficient can indicate linearity, it does not guarantee it. Therefore, data on the correlation coefficients was augmented with the relative standard deviation (RSD). To confirm linearity, the RSD should be < 5% (26). Based on the RSD criterion, none of the compounds exhibited a linear response throughout the entire concentration range. Most compounds showed the best linearity at the lower concentration range, except for hexanal, hexanoic acid and octanoic acid, which displayed the best linearity at the higher concentration range (0.63–16.2, 1.35–18.0, and 1.19–19.1 mg kg^−1^, respectively). One-octanol, octanal and benzene, 1,4-dimethoxy showed the best linearity at the lower range of the concentrations (0.07–0.91 mg kg^−1^). The LOD varied between 0.03 and 1.13 mg kg^−1^. Octanal and benzaldehyde had the lowest LODs (0.03 mg kg^−1^), while octanoic acid had the highest (1.13 mg kg^−1^). The working range was determined as LOQ to the highest concentration tested in the linear range. Recovery ranged from 94.2% (1-octen-3-ol) to 106% (octanoic acid) for the volatiles in the dry-cured ham.

**Table 3:** Validation parameters.

**No**	**Compound**	***R***	**RSD (%)**	**Recovery (%)**	**Calibration curve**	**LOD**	**LOQ**	**WR**	**ISTD**	***R*_ISTD_**
1	1-Octanol	0.996	4.56	98.7	*y* = 5E+07 *x* + 914,709	0.07	0.22	0.09–0.91	Linalool	0.998
2	1-Octen-3-ol	0.992	4.70	94.2	*y* = 5E+07 *x* + 1E+06	0.11	0.34	0.08–1.28	2-Hexen-1-ol, *(E)-*	0.995
3	Hexanal	0.996	2.28	101	*y* = 1E+06 *x* + 864,040	0.85	2.56	0.63–16.2	Toluene-d8	0.995
4	Heptanal	0.993	7.45	98.8	*y* = 1E+07 *x* + 37,931	0.12	0.36	0.01–1.40	Butanoic acid, butyl ester	0.999
5	Octanal	0.996	10.8	102	*y* = 1E+07 *x* + 16,464	0.03	0.09	0.01–0.73	Acetic acid, pentyl ester	0.996
6	Nonanal	0.992	7.87	101	*y* = 2E+07 *x* + 403,408	0.12	0.36	0.09–1.38	2-Hexen-1-ol, acetate, *(E)*-	0.995
7	2-Decenal, *(E)-*	0.996	5.84	104	*y* = 4E+06 *x* + 878,858	0.61	1.83	0.08–10.4	Linalool	0.998
8	Benzaldehyde	0.999	4.49	101	*y* = 9E+07 *x* + 88,940	0.03	0.09	0.01–1.05	Linalool	0.996
9	Hexanoic acid	0.996	4.68	102	*y* = 5E+06 *x* – 5E+06	0.90	2.73	1.35–18.0	γ-Dodecalactone	0.999
10	Octanoic acid	0.997	6.71	106	*y* = 1E+07 *x* – 2E+06	1.13	3.41	1.19–19.1	γ-Dodecalactone	0.998
11	Dodecanoic acid	0.999	4.14	99.6	*y* = 8E+06 *x* – 4E+06	0.15	0.46	0.65–3.77	γ-Dodecalactone	0.993
12	Benzene, 1,4-dimethoxy	0.992	5.00	99.2	*y* = 1E+08 *x* + 3E+06	0.06	0.18	0.07–0.70	Linalool	0.998

R, squared adjusted regression coefficient; RSD, relative standard deviation; LOD, limits of detection (mg kg^−1^); LOQ, limits of quantification (mg kg^−1^); WR, working range (mg kg^−1^); ISTD, internal standards; *R*_ISTD_, squared adjusted regression coefficient of internal standard.

#### 3.2.1 Multiple internal standard addition for improving HS-SPME/GC-MS quantitation

Table 3 presents information regarding the selection of the most suitable ISTDs [2-hexen-1-ol, *(E)*; 2-hexen-1-ol, acetate, *(E)*; 3-heptanone; 2-methyl; acetic acid, pentyl ester; acetic acid, hexyl ester; butanoic acid, methyl ester; butanoic acid, ethyl ester; butanoic acid, butyl ester; hexanoic acid, ethyl ester; linalool; γ-dodecalactone and toluene-d_8_] for 12 selected compounds found in the aromatic profile of dry-cured ham. These internal standards were selected based on chromatographic retention time (rt), a wide linear working range, linear correlation, an intercept close to zero and chemical class similarity. These criteria ensured that the selected internal standards were distributed throughout the chromatogram and did not overlap with external standards. Each of these 12 analytes was calibrated by choosing the most suitable ISTD.

**Figure 6:**
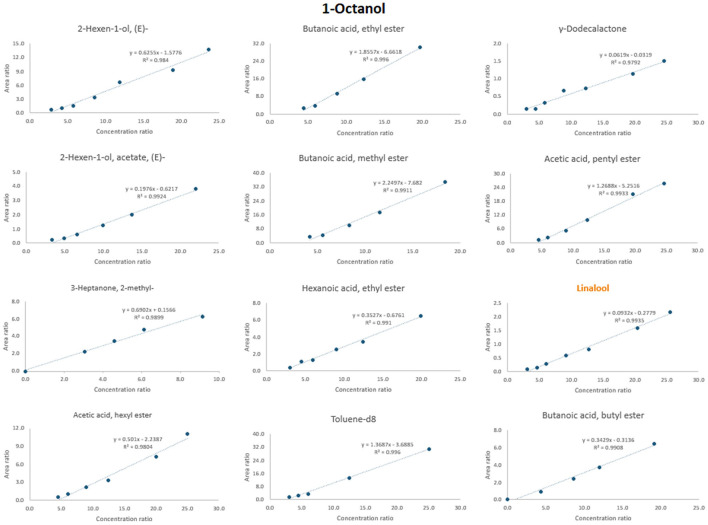
Procedure of the selection of the most appropriate ISTD for 1-octanol. The name of the chosen ISTD is marked with orange color.

Figure 6 shows an example of selecting an appropriate internal standard for the compound 1-octanol. When considering the adjusted regression coefficient (*R*^2^), several internal standards exhibited values close to 0.99, including 2-hexen-1-ol, acetate, ***(E)***; acetic acid, pentyl ester; butanoic acid, methyl ester; butanoic acid, ethyl ester; butanoic acid, butyl ester; hexanoic acid, ethyl ester; linalool; and toluene-d_8_. Also, when retention time is considered, compounds such as linalool (rt = 14.0) and 2-hexen-1-ol, acetate, ***(E)*** (rt = 9.5) closely match that of 1-octanol (rt = 14.3). Further linalool is also justified as the suitable internal standard due to its nearly zero intercept.

The same principle was applied to all other analytes. For instance, compounds such as linalool, γ-dodecalactone, toluene-d_8_, 2-hexen-1-ol, *(E)*; 2-hexen-1-ol, acetate, *(E)*; butanoic acid, butyl ester and acetic acid, pentyl ester were found to be the most commonly used ISTDs (Table 3). Supplementary Figure 1 shows the calibration curves for all 12 analytes selected from different classes (e.g., alcohols, aldehydes and acids). From each class, the most appropriate ISTD was chosen.

### 3.3 Volatile organic compound profile of four dry-cured hams—Preliminary study

**Figure 7:**
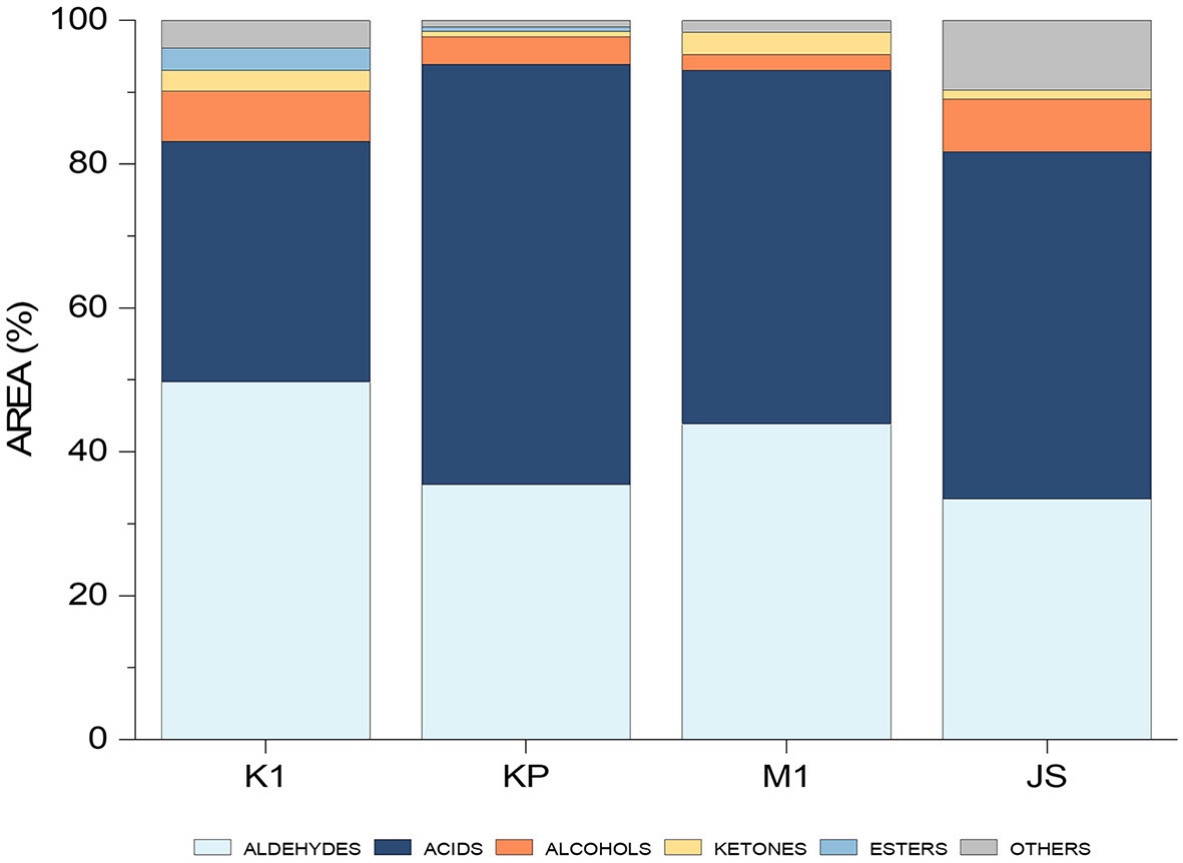
Histogram showing the differentiation between individual dry-cured ham samples (K1, Kraški pršut; KP, dry-cured ham from Kraškopolje pig; M1, dry-cured ham made from pork of the Mangalica variety; JS, Spanish Jamon Serrano) based on their aromatic profiles determined by the HS-SPME/GC-MS.

The optimized and validated method was then used on the real samples, including four dry-cured ham samples from the Slovenian market, namely Kraški pršut (K1), Kraškopolje pig (KP), Mangalica (M1), and Jamon Serrano (JS). Peak areas were summed and presented as a percentage of the total VOCs in each sample (Figure 7). The predominant chemical groups included aldehydes, acids, alcohols, ketones, and esters.

Aldehydes are represented in all samples originating mainly from fatty acid autoxidation (linear aldehydes) and amino acid degradation (branched and aromatic aldehydes) (1). Sensory descriptors associated with linear aldehydes include grassy, green, fatty, fresh (40), meat-like, ham-like, and rancid (5). The occurrence of acids in the samples can be attributed to triglyceride and phospholipid hydrolysis or the oxidation of unsaturated fatty acids (1), possibly exacerbated by enzymatic lipolysis during ham ripening (5). Short-chain acids, with their low perception threshold, are significant contributors to the overall aroma. On the other hand, long-chain acids like octanoic, nonanoic and decanoic acids, which have higher odor thresholds, do not significantly influence the overall aroma. However, these long-chain acids are probably precursors for other odor-active compounds, i.e., aldehydes, alcohols, ketones, and shorter-chain carboxylic acids, which are produced during the ripening stage (1).

Alcohols, both linear and branched, present in the samples and resulting from lipid oxidation, are regarded as minor contributors to the overall aroma due to their higher odor thresholds (1). Their sensory notes include herbaceous, woody, fatty (5), sweet, fruity and mushroom (32). However, certain alcohols, particularly straight-chain unsaturated ones (e.g., 1-octen-3-ol), have lower thresholds and thus their impact on aroma may be considerable (41). Ketones are also present, and although their origin is diverse, they are most commonly formed from the oxidation of lipids. Nevertheless, the formation of these compounds can occur through the Maillard reaction as well as through the microorganism-inducted esterification. These compounds significantly influence the aroma profile of meat products. Additionally, in lower concentrations, they are capable of producing buttery, blue cheese, and spicy notes (34, 40) while also being linked with aromas typical of cooked meat (5).

**Figure 8:**
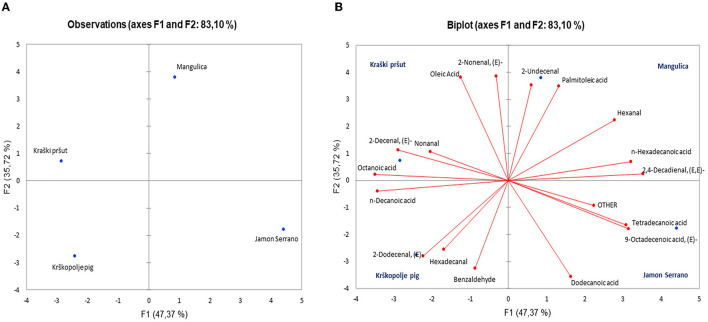
Principal component analysis (PCA) of samples of dry-cured ham samples (Kraški pršut, Krškopolje pig, Mangulica, and Jamon Serrano): **(A)** discriminant function score plot, and **(B)** discriminant loading plot, showing correlations between initial variables and the discriminant functions for the different dry-cured hams.

Principal Component Analysis (PCA) was applied to determine whether it can differentiate between dry-cured hams based on their VOC profiles. Figure 8A presents a score plot that distributes various ham types (Mangulica, Kraški pršut, Krškopolje pig, Jamon Serrano) across the first two principal components, F1 and F2, which account for 47.37 and 35.72% of the variance, respectively, summing up to 83.10% of the total variance. This suggests differences in the variables represented by F1 and F2 among the ham samples. Figure 8B, a biplot, illustrates these observations along with the variables (e.g., aldehydes, acids) influencing their flavor profiles, where the proximity of volatile compounds to specific ham types indicates a stronger association with their aroma profiles. The results showed that it is possible to distinguish between dry-cured hams based on their aromatic profiles. For instance, the discriminating compounds in Kraški pršut included oleic acid, octanoic acid, 2-decenal *(E)*, 2-nonenal *(E)*, and nonanal. Compounds, such as 2-dodecenal *(E), n-*decanoic acid, benzaldehyde, and hexadecanal, contributed to the differentiation of krškopolje pig dry-cured ham. For Mangulica, the discriminative compounds were 2,4-decadienal *(E,E)*, palmitoleic acid, 2-undecenal, *n-*hexadecanoic acid, and hexanal. In Jamon Serrano, key compounds were acids: 9-octadecanoic acid *(E)*, tetradecanoic acid, and dodecanoic acid. It seems that dry-hams except Kraški pršut exhibit higher content of interamuscular fat. However, it is important to note that this study is preliminary, involving only four samples, so it is difficult to conclude why specific VOCs are important for ham differentiation. Nevertheless, the study gives the encouraging results and the developed method will be applied to a much larger sample set in the future.

## 4 Conclusions

In this study, we successfully optimized and validated an analytical method for determining VOCs in dry-cured ham based on a HS-SPME combination with GC-MS. An optimal SPME method was obtained using a one-factor-at-a-time approach and RSM. Based on the results obtained from both methods, the optimal SPME conditions were 60 min of equilibration, 60 min of extraction at 70°C, and a desorption time of 4 min.

In a preliminary study involving four samples from the Slovenian market, the method could differentiate all four hams based on specific VOCs, highlighting the method's potential. While this study provides valuable insights into VOC analysis in dry-cured ham, it is essential to emphasize its preliminary nature, limited to small collected samples. Further investigations involving more samples are requisite. Nevertheless, this experimental approach offers a robust means of detecting volatile compounds in dry-cured ham, contributing to a more comprehensive appreciation of their sensory properties.

## Data availability statement

The original contributions of this study are included in the article/Supplementary material and are available at: DOI: 10.5281/zenodo.10469682, while further inquiries can be directed to the corresponding author.

## Author contributions

KB: Conceptualization, Data curation, Formal analysis, Investigation, Methodology, Validation, Visualization, Writing – original draft. LS: Conceptualization, Data curation, Formal analysis, Methodology, Validation, Writing – review & editing. AĆ: Data curation, Methodology, Visualization, Writing – review & editing. NO: Conceptualization, Funding acquisition, Investigation, Resources, Supervision, Validation, Writing – review & editing.

## References

[1] Sirtori F Dimauro C Bozzi R Aquilani C Franci O Calamai L. Evolution of volatile compounds and physical, chemical and sensory characteristics of Toscano PDO ham from fresh to dry-cured product. Eur Food Res Technol. (2020) 246:409–24. 10.1007/s00217-019-03410-0

[2] Liu H Huang J Hu Q Chen YP Lai K Xu J. Dual-fiber solid-phase microextraction coupled with gas chromatography–mass spectrometry for the analysis of volatile compounds in traditional Chinese dry-cured ham. J Chromatogr B Anal Technol Biomed Life Sci. (2020) 1140:121994. 10.1016/j.jchromb.2020.121994 32028114

[3] Škrlep M Candek-Potokar M Lukač NB Povše MP Pugliese C Labussière E. Comparison of entire male and immunocastrated pigs for dry-cured ham production under two salting regimes. Meat Sci. (2016) 111:27–37. 10.1016/j.meatsci.2015.08.010 26331963

[4] Pugliese C Sirtori F Škrlep M Piasentier E Calamai L Franci O. The effect of ripening time on the chemical, textural, volatile and sensorial traits of Bicep femoris and Semimembranosus muscles of the Slovenian dry-cured ham Kraški pršut. Meat Sci. (2015) 100:58–68. 10.1016/j.meatsci.2014.09.012 25306512

[5] García-González DL Aparicio R Aparicio-Ruiz R. Volatile and amino acid profiling of dry cured hams from different swine breeds and processing methods. Molecules. (2013) 18:3927–47. 10.3390/molecules18043927 23552905 PMC6270080

[6] Martínez-Onandi N Rivas-Cañedo A Picon A Nuñez M. Influence of physicochemical parameters and high pressure processing on the volatile compounds of Serrano dry-cured ham after prolonged refrigerated storage. Meat Sci. (2016) 122:101–8. 10.1016/j.meatsci.2016.07.027 27513944

[7] Domínguez R Purriños L Pérez-Santaescolástica C Pateiro M Barba FJ Tomasevic I. Characterization of volatile compounds of dry-cured meat products using HS-SPME-GC/MS technique. Food Anal Methods. (2019) 12:1263–84. 10.1007/s12161-019-01491-x

[8] Xu CH Chen GS Xiong ZH Fan YX Wang XC Liu Y. Applications of solid-phase microextraction in food analysis. Trends Anal Chem. (2016) 80:12–29. 10.1016/j.trac.2016.02.02210890509

[9] Paiva AC Crucello J de Aguiar Porto N Hantao LW. Fundamentals of and recent advances in sorbent-based headspace extractions. Trends Anal Chem. (2021) 139:116252. 10.1016/j.trac.2021.116252

[10] Karabagias IK. Volatile profile of raw lamb meat stored at 4 ± 1 °C: the potential of specific aldehyde ratios as indicators of lamb meat quality. Foods. (2018) 7:1–11. 10.3390/foods7030040 29547528 PMC5867555

[11] Muñoz-Redondo JM Ruiz-Moreno MJ Puertas B Cantos-Villar E Moreno-Rojas JM. Multivariate optimization of headspace solid-phase microextraction coupled to gas chromatography-mass spectrometry for the analysis of terpenoids in sparkling wines. Talanta. (2019) 208:120483. 10.1016/j.talanta.2019.120483 31816799

[12] Anil I Öztürk N Alagha O Ergenekon P. Optimization of solid-phase microextraction using Taguchi design to quantify trace level polycyclic aromatic hydrocarbons in water. J Sep Sci. (2012) 35:3561–8. 10.1002/jssc.201200550 23225720

[13] Jalili V Barkhordari A Ghiasvand A. A comprehensive look at solid-phase microextraction technique: a review of reviews. Microchem J. (2020) 152:104319. 10.1016/j.microc.2019.104319

[14] Xu XB Murtada K Pawliszyn J. Determination of selected volatile terpenes in fish samples via solid phase microextraction arrow coupled with GC-MS. Talanta. (2021) 221:121446. 10.1016/j.talanta.2020.121446 33076070

[15] Mohammadhosseini M. Chemical composition of the essential oils and volatile fractions from flowers, stems and roots of *Salvia multicaulis* Vahl. by using MAHD, SFME and HS-SPME methods. J Essent Oil Bear Plants. (2015) 18:1360–71. 10.1080/0972060X.2015.1024447

[16] Hough R Archer D Probert C. A comparison of sample preparation methods for extracting volatile organic compounds (VOCs) from equine faeces using HS-SPME. Metabolomics. (2018) 14:1–10. 10.1007/s11306-017-1315-7 29367839 PMC5754382

[17] Ma QL Hamid N Bekhit AED Robertson J Law TF. Optimization of headspace solid phase microextraction (HS-SPME) for gas chromatography mass spectrometry (GC–MS) analysis of aroma compounds in cooked beef using response surface methodology. Microchem J. (2013) 111:16–24. 10.1016/j.microc.2012.10.007

[18] Bezerra MA Santelli RE Oliveira EP Villar LS Escaleira LA. Response surface methodology (RSM) as a tool for optimization in analytical chemistry. Talanta. (2008) 76:965–77. 10.1016/j.talanta.2008.05.019 18761143

[19] Ilaiyaraja N Likhith KR Sharath Babu GR Khanum F. Optimisation of extraction of bioactive compounds from Feronia limonia (wood apple) fruit using response surface methodology (RSM). Food Chem. (2015) 173:348–54. 10.1016/j.foodchem.2014.10.035 25466032

[20] Yolmeh M Jafari SM. Applications of response surface methodology in the food industry processes. Food Bioprocess Technol. (2017) 10:413–33. 10.1007/s11947-016-1855-2

[21] Rambla-Alegre M Esteve-Romero J Carda-Broch S. Is it really necessary to validate an analytical method or not? That is the question. J Chromatogr A. (2012) 1232:101–9. 10.1016/j.chroma.2011.10.050 22099221

[22] Fortini M Migliorini M Cherubini C Cecchi L Calamai L. Multiple internal standard normalization for improving HS-SPME-GC-MS quantitation in virgin olive oil volatile organic compounds (VOO-VOCs) profile. Talanta. (2017) 165:641–52. 10.1016/j.talanta.2016.12.082 28153311

[23] Petričević S Marušić Radovčić N Lukić K Listeš E Medić H. Differentiation of dry-cured hams from different processing methods by means of volatile compounds, physico-chemical and sensory analysis. Meat Sci. (2018) 137:217–27. 10.1016/j.meatsci.2017.12.001 29223014

[24] D'Agostino MF Sanz J Martínez-Castro I Giuffrè AM Sicari V Soria AC. Statistical analysis for improving data precision in the SPME GC–MS analysis of blackberry (*Rubus ulmifolius* Schott) volatiles. Talanta. (2014) 125:248–56. 10.1016/j.talanta.2014.02.058 24840441

[25] Strojnik L Hladnik J Weber NC Koron D Stopar M Zlatić E. Construction of isovoc database for the authentication of natural flavours. Foods. (2021) 10:1550. 10.3390/foods10071550 34359420 PMC8306145

[26] Šiškovič N Strojnik L Grebenc T Vidrih R Ogrinc N. Differentiation between species and regional origin of fresh and freeze-dried truffles according to their volatile profiles. Food Control. (2021) 123:107698. 10.1016/j.foodcont.2020.107698

[27] Mohammadhosseini M Akbarzadeh A Flamini G. Profiling of compositions of essential oils and volatiles of salvia limbata using traditional and advanced techniques and evaluation for biological activities of their extracts. Chem Biodivers. (2017) 14. 10.1002/cbdv.201600361 28273408

[28] Strojnik L Grebenc T Ogrinc N. Species and geographic variability in truffle aromas. Food Chem Toxicol. (2020) 142:111434. 10.1016/j.fct.2020.111434 32442473

[29] Van Den Dool H Kratz PD. A generalization of the retention index system including linear temperature programmed gas-liquid partition chromatography. J Chromatogr A. (1963) 11:463–71. 10.1016/S0021-9673(01)80947-X 14062605

[30] Arcari SG Caliari V Sganzerla M Godoy HT. Volatile composition of Merlot red wine and its contribution to the aroma: optimization and validation of analytical method. Talanta. (2017) 174:752–66. 10.1016/j.talanta.2017.06.074 28738652

[31] Mohammadhosseini M. Chemical composition of the volatile fractions from flowers, leaves and stems of *Salvia mirzayanii* by HS-SPME-GC-MS. J Essent Oil Bear Plants. (2015) 18:464–76. 10.1080/0972060X.2014.1001185

[32] Bosse (née Danz) R Wirth M Konstanz A Becker T Weiss J Gibis M. Determination of volatile marker compounds in raw ham using headspace-trap gas chromatography. Food Chem. (2017) 219:249–59. 10.1016/j.foodchem.2016.09.094 27765224

[33] Moon SY Li-Chan ECY. Development of solid-phase microextraction methodology for analysis of headspace volatile compounds in simulated beef flavour. Food Chem. (2004) 88:141–9. 10.1016/j.foodchem.2004.04.002

[34] Li H Geng W Haruna SA Zhou C Wang Y Ouyang Q. Identification of characteristic volatiles and metabolomic pathway during pork storage using HS-SPME-GC/MS coupled with multivariate analysis. Food Chem. (2022) 373:131431. 10.1016/j.foodchem.2021.131431 34700034

[35] Drakula S Mustač NC Novotni D Voučko B Krpan M Hruškar M. Optimization and validation of a HS-SPME/GC–MS method for the analysis of gluten-free bread volatile flavor compounds. Food Anal Methods. (2022) 15:1155–70. 10.1007/s12161-021-02076-3

[36] Arvindekar AU Laddha KS. An efficient microwave-assisted extraction of anthraquinones from Rheum emodi: optimisation using RSM, UV and HPLC analysis and antioxidant studies. Ind Crops Prod. (2016) 83:587–95. 10.1016/j.indcrop.2015.12.066

[37] Prasad KN Hassan FA Yang B Kong KW Ramanan RN Azlan A. Response surface optimisation for the extraction of phenolic compounds and antioxidant capacities of underutilised Mangifera pajang Kosterm. peels Food Chem. (2011) 128:1121–7. 10.1016/j.foodchem.2011.03.105

[38] Belwal T Dhyani P Bhatt ID Rawal RS Pande V. Optimization extraction conditions for improving phenolic content and antioxidant activity in Berberis asiatica fruits using response surface methodology (RSM). Food Chem. (2016) 207:115–24. 10.1016/j.foodchem.2016.03.081 27080887

[39] Souza-Silva ÉA Gionfriddo E Pawliszyn J A. critical review of the state of the art of solid-phase microextraction of complex matrices II. Food analysis. Trends Anal Chem. (2015) 71:236–48. 10.1016/j.trac.2015.04.018

[40] Merlo TC Lorenzo JM Saldaña E Patinho I Oliveira AC Menegali BS. Relationship between volatile organic compounds, free amino acids, and sensory profile of smoked bacon. Meat Sci. (2021) 181:108596. 10.1016/j.meatsci.2021.108596 34118571

[41] Pugliese C Sirtori F Calamai L Franci O. The evolution of volatile compounds profile of “Toscano” dry-cured ham during ripening as revealed by SPME-GC-MS approach. J Mass Spectrom. (2010) 45:1056–64. 10.1002/jms.1805 20799283

