# Supplementary material for: Optimization and validation of an HS-SPME/GC-MS method for determining volatile organic compounds in dry-cured ham
Source: Front Nutr. 2024 Feb 1;11:1342417. doi: 10.3389/fnut.2024.1342417 (PMC10867123; doi:10.3389/fnut.2024.1342417)
Supplement: Supplementary file 1 [file Table_1.DOCX]

Supplementary Material

Optimization and validation of an HS-SPME/GC-MS method for determining of volatile organic compounds (VOCs) in dry-cured ham

Katja Babič^1,2^, Lidija Strojnik^2^, Andrija Ćirić^3^, Nives Ogrinc^1,2*^

^1^Jožef Stefan International Postgraduate School, Ljubljana, Slovenia

^2^Department of Environmental Sciences, Jožef Stefan Institute, Ljubljana, Slovenia

^3^University of Kragujevac, Faculty of Science, Department of Chemistry, Serbia

*** Correspondence:** Nives Ogrinc; [nives.ogrinc@ijs.si](mailto:nives.ogrinc@ijs.si)

# Supplementary Table

**TABLE 1:** Results of the experimentally measured responses for the 15 runs according to the experimental design.

| **Run** | **Equilibrium time (min)** | **Extraction time (min)** | **Temperature (°C)** | **Response average (×10^6^)** | **Total aldehyde**  **(×10^6^)** | **Total acids (×10^6^)** |
| --- | --- | --- | --- | --- | --- | --- |
| **1** | **20.0** | **20.0** | **70.0** | **3.299** | **0.878** | **10.11** |
| **2** | **40.0** | **40.0** | **50.0** | **1.288** | **0.164** | **1.868** |
| **3** | **40.0** | **40.0** | **60.0** | **2.896** | **0.369** | **4.298** |
| **4** | **60.0** | **20.0** | **50.0** | **0.924** | **1.537** | **0.528** |
| **5** | **20.0** | **60.0** | **70.0** | **6.371** | **5.328** | **12.65** |
| **6** | **40.0** | **20.0** | **60.0** | **1.229** | **1.823** | **1.475** |
| **7** | **20.0** | **60.0** | **50.0** | **1.363** | **1.817** | **1.938** |
| **8** | **20.0** | **20.0** | **50.0** | **0.787** | **1.168** | **0.903** |
| **9** | **20.0** | **40.0** | **60.0** | **1.734** | **1.671** | **3.721** |
| **10** | **40.0** | **60.0** | **60.0** | **3.633** | **3.891** | **7.166** |
| **11** | **60.0** | **60.0** | **70.0** | **7.852** | **4.801** | **19.11** |
| **12** | **60.0** | **20.0** | **70.0** | **2.230** | **1.963** | **4.269** |
| **13** | **60.0** | **60.0** | **50.0** | **1.230** | **2.247** | **0.609** |
| **14** | **60.0** | **40.0** | **60.0** | **2.905** | **3.454** | **4.997** |
| **15** | **40.0** | **40.0** | **70.0** | **3.518** | **1.931** | **8.942** |

# Supplementary Figure

**Supplementary Figure 1.** Selection of the most appropriate ISTD for twelve compounds: 1-octanol; 1-octen-3-ol; hexanal; heptanal; octanal; nonenal; 2-decenal, (E); benzaldehyde; hexanoic acid; octanoic acid; dodecanoic acid, and benzene, 1,4-dimethoxy. The name of the chosen ISTD is marked with black colour, below the specific compound.
